# Diacerein counteracts acetaminophen-induced hepatotoxicity in mice via targeting NLRP3/caspase-1/IL-1β and IL-4/MCP-1 signaling pathways

**DOI:** 10.1007/s12272-022-01373-7

**Published:** 2022-03-04

**Authors:** Mahmoud Elshal, Marwa E. Abdelmageed

**Affiliations:** Pharmacology and Toxicology Department, Faculty of Pharmacy, Mansoura University, El Gomhoria Street, Eldakahlia, 35516 Egypt

**Keywords:** Diacerein, Acetaminophen, NLRP3, IL-1β, IL-4, MCP-1

## Abstract

The current study aims at repurposing the anti-arthritic drug diacerein (DCN) for the treatment of acetaminophen hepatotoxicity and investigating the potential underlying mechanisms. Mice were randomly divided into six groups receiving either no treatment (control group), 20 mg/kg DCN *i.p*, 400 mg/kg acetaminophen *i.p*, DCN 4 h before acetaminophen, DCN 2 h after acetaminophen, or 400 mg/kg N-acetylcysteine (NAC) *i.p*, 2 h after acetaminophen. Biomarkers of liver dysfunction, oxidative stress, and apoptosis were assessed. Hepatic necroinflammatory changes were evaluated along with hepatic expression of NF-κB and caspase-1. The levels of NLRP3, IL-1β, IL-4, MCP-1, and TNF-α in the liver, as well as CYP2E1 mRNA expression, were measured. Diacerein significantly reduced biomarkers of liver dysfunction, oxidative stress, hepatocyte necrosis, and infiltration of neutrophils and macrophages whether administered 4 h before or 2 h after acetaminophen. Further, the effects were comparable to those of NAC. Diacerein also counteracted acetaminophen-induced hepatocellular apoptosis by increasing Bcl-2 and decreasing Bax and caspase-3 expression levels. Moreover, DCN normalized hepatic TNF-α and significantly decreased NF-κB p65 expression. Accordingly, DCN can prevent or reverse acetaminophen hepatotoxicity in mice, suggesting potential utility as a repurposed drug for clinical treatment.

## Introduction

Drug-induced hepatotoxicity is a potentially life-threatening adverse drug reaction responsible for the majority of acute liver failure cases and a leading reason for liver transplantation (Khoury et al. 2015).

Acetaminophen (Paracetamol, N-acetyl-p-aminophenol) is among the most frequently used analgesic and antipyretic drugs. Although it is safe at recommended doses, overdose may result in hepatotoxicity and acute liver failure (Yan et al. 2018; Simões et al. 2019).

Acetaminophen hepatotoxicity involves multiple stages and pathogenic mechanisms, including the generation of toxic metabolite, oxidative stress, mitochondrial dysfunction, and sterile inflammation, ultimately leading to hepatocyte death (Yan et al. 2018; Mary and Ezhilarasan 2020). Acute acetaminophen hepatotoxicity is initiated by the highly reactive N-acetyl-p-benzoquinoneimine (NAPQI), which is generated mainly by cytochrome P450 2E1 (CYP2E1)-mediated metabolism (Karthivashan et al. 2015). It is believed that during acetaminophen overdose, NAPQI detoxification by conjugation to the endogenous antioxidant glutathione (GSH) depletes the cellular GSH pool, reducing the activities of GSH-dependent antioxidant enzymes. Further, the remaining NAPQI can covalently bind to liver proteins, generating cytotoxic arylated proteins. In combination, these processes initiate oxidative stress that leads to hepatocyte death, mainly via necrosis (Lee et al. 1996; Athersuch et al. 2018).

Necrotic hepatocytes release damage-associated molecular patterns (DAMPs) that activate the NLR family pyrin domain containing 3 (NLRP3) inflammasome, which cleaves procaspase-1, forming active caspase-1 (Roh and Sohn 2018; Li et al. 2021). Caspase-1 then cleaves pro-interleukin-1 beta (pro-IL-1β), forming active IL-1β, which triggers the recruitment of neutrophils and monocytes to the liver (Woolbright and Jaeschke 2017; Li et al. 2021; Seok et al. 2021).

Liver injury may also be associated with apoptotic cell death. Apoptosis is regulated primarily by B-cell leukemia/lymphoma 2 (Bcl-2) family members, including both anti-apoptotic Bcl-2 and pro-apoptotic Bcl2 associated X (Bax). Bcl-2 and Bax reciprocally regulate the downstream effector caspase-3, which in turn executes apoptosis via the targeted cleavage of cellular proteins involved in DNA fragmentation, cytoskeletal breakdown, and various other catabolic processes (Oz and Chen 2008; Kitazumi and Tsukahara 2011; Martinou and Youle 2011).

Other cytokines may orchestrate the liver response to injury, such as tumor necrosis factor alpha (TNF-α) (Tilg et al. 2006). However, there are still uncertainties regarding the signaling pathways that underlie acetaminophen hepatotoxicity. Two outstanding uncertainties are the contributions of the NLRP3 inflammasome and IL-4 signaling pathways (Yoon et al. 2016; Jaeschke 2018; Shi et al. 2020).

There is currently a growing attention to repurpose FDA-approved drugs, especially those of natural origin, for other conditions as this strategy is faster and much less expensive than traditional drug development and likely safer as approved drugs have already been extensively tested for side-effects. Diacerein (DCN) is an anthraquinone approved by the FDA as an anti-inflammatory for symptomatic treatment of osteoarthritis. Clinical effects are mediated at least in part by the active metabolite rhein, which has demonstrated anti-inflammatory properties (Almezgagi et al. 2020).

This present study investigated the potential preventive and curative effects of DCN on acetaminophen hepatotoxicity in mice to provide a potential new drug choice with multimodal actions. The current study demonstrates that DCN can prevent or mitigate hepatotoxicity in mice by reducing oxidative stress, inflammation, necrosis, and apoptosis induced by acetaminophen overdose.

## Materials and methods

### Animals

Adult BALB/c mice weighing (25–35 g) were obtained from VACSERA [Agouza, Giza, Egypt] and housed in a controlled environment maintained at 25 °C under a 12 h/12 h light/dark cycle with free access to food and water. All care and experimental protocols were approved by the research ethics committee of Mansoura University, Mansoura, Egypt (code number: 2021-267).

### Drugs and chemicals

Acetaminophen was purchased as an injectable solution (Perfalgan, 10 mg/mL, Bristol‐Myers Squibb, Victoria, Australia). NAC was purchased as the pharmaceutical drug (Fluimucil, 100 mg/mL, The Cathay Drug Company Inc., Makati, Philippines). DCN was purchased as a pure powder from Sigma-Aldrich (Saint Louis, MO, USA), dissolved in 0.5% carboxymethylcellulose, and further diluted in 0.01 M phosphate-buffered saline (PBS) to a final concentration of 4 mg/mL. All other chemicals used in this study were of high analytical grade.

### Experimental design

The doses of acetaminophen and NAC were chosen based on our pilot study that is consistent with previous mouse studies using acetaminophen (Yoshioka et al. 2017; Zhang et al. 2017) or NAC (Owumi et al. 2015). The DCN dose (20 mg/kg) was also chosen based on previous studies (Douni et al. 2004; Tobar et al. 2011; Paulino et al. 2020) and a pilot study testing the effects of 5, 10, and 20 mg/kg injections 4 h before acetaminophen injection on serum biomarkers of liver dysfunction. The 20 mg/kg dose was chosen as it achieved the maximum protective response without inducing liver dysfunction when administered alone.

Mice were fasted for 12 h with free access to water before acetaminophen administration to create comparable conditions for acetaminophen metabolism. Immediately after the fasting period, acetaminophen was administered at 400 mg/kg body weight, and then free access to food (standard chow diet) was allowed (Mossanen and Tacke 2015). The total experimental period was 36 h.

Mice were randomly divided into the following six groups of eight animals: Group I (control), animals received vehicle without DCN; Group II (DCN), animals received DCN (20 mg/kg) intraperitoneally (*i.p.*); Group III (acetaminophen), animals received acetaminophen (400 mg/kg) *i.p.*; Group IV (DCN 4 h prior to acetaminophen), animals received DCN (20 mg/kg) *i.p*. followed by acetaminophen (400 mg/kg) *i.p*. 4 h later; Group V (DCN 2 h post-acetaminophen), animals received acetaminophen (400 mg/kg) *i.p.* followed by DCN (20 mg/kg) *i.p* 2 h later; Group VI (NAC 2 h post-acetaminophen), animals received acetaminophen (400 mg/kg) followed by NAC (400 mg/kg) *i.p.* 2 h later.

### Sample collection and preparation

Twenty-four hours after acetaminophen injection, mice were anesthetized by thiopental (70 mg/kg *i.p.*) (Elshal et al. 2019). Blood samples were withdrawn from the heart and centrifuged at 3000 g for 10 min at 4 °C to isolate serum, which was stored at − 80 °C for further analysis. The right median lobe of the liver was fixed in 10% (v/v) neutral-buffered formalin for immunohistochemical and histopathological assessment. A part of the left median lobe was homogenized in PBS (10% w/v) and centrifuged at 3000 g at 4 °C for 15 min, and the supernatant was stored at − 80 °C for biochemical measurements and enzyme-linked immunosorbent assays (ELISAs). Another part of the left median lobe was flash-frozen in liquid nitrogen and stored at − 80 °C for quantitative RT-PCR.

### Liver function biomarkers

Serum alanine aminotransferase (ALT) and aspartate aminotransferase (AST) activities were assessed as markers of liver injury using commercial kits (BIODIAGNOSTIC, Giza, Egypt) according to the manufacturer’s instructions. Serum lactate dehydrogenase (LDH) activity was determined using a commercial kit (Human diagnostics, Wiesbaden, Hesse, Germany).

### Hepatic histopathological evaluation

Pre-fixed liver tissues were processed into paraffin blocks followed by preparing 5 μm sections mounted on slides and staining with hematoxylin–eosin (H&E) for hepatic histopathological assessment. Histopathological lesions in H&E-stained hepatic specimens were examined for the presence of necrosis and inflammation. As for necrosis; grade 0: lesions occupied less than 5% of the hepatic parenchyma; grade 1: lesions occupied 6–33% of the hepatic parenchyma; grade 2: lesions occupied 34–66% of the hepatic parenchyma, and grade 3: lesions occupied more than 66% of the hepatic parenchyma. Inflammatory cell infiltration was graded as follows: grade 0, no infiltration; grade 1, one to two foci per 100 × field; grade 2, three to four foci per 100 × field; grade 3, more than four foci per 100 × field.

### Hepatic oxidative stress and antioxidant parameters

Reduced glutathione (GSH) was estimated based on the method of Ellman that was used to measure non-protein sulfhydryl compound using trichloro acetic acid-deproteinized tissue supernatant (Ellman 1959). Enzymatic antioxidants; glutathione reductase (GR), glutathione peroxidase (GPx), and glutathione-S-transferase (GST) activities were estimated in hepatic homogenates using commercially kits (BIODIAGNOSTIC, Giza, Egypt).

Superoxide dismutase (SOD) activity was assessed by detecting the degree of inhibition of the auto-oxidation of pyrogallol by SOD (Marklund and Marklund 1974). Malondialdehyde (MDA), the lipid peroxidation end product, was assessed by measurement thiobarbituric acid reactive substances according to the method of Ohkawa et al. (Ohkawa et al. 1979).

### Immunohistochemical examination

The expression levels of F4/80, nuclear Factor Kappa B (NF-κB) p65, and caspase-1 in hepatic tissues were estimated by immunostaining using the Avidin‐Biotin Complex method (Guesdon et al. 1979) and the following antibodies: F4/80 mouse monoclonal antibody (Cat. No: 123101; BioLegend, CA, USA), NF-κB p65 mouse monoclonal antibody, and caspase-1 mouse monoclonal antibody (Cat. No: PA5-29342 and PA5-27617, respectively, Thermo Fisher Scientific Anatomical Pathology, CA, USA).

### Assessment of cytokines and inflammatory mediators

Monocyte chemoattractant protein-1 (MCP-1) and NLRP3 levels were assessed in hepatic homogenate using commercial ELISA Kits from Boster Biological Technology (CA, USA) and LifeSpan Biosciences (CA, USA), respectively.

Hepatic levels of TNF‐α, IL-1β, IL‐4, and IL-10 were also assessed using ELISA kits from R&D Systems Inc. (MN, USA) according to the manufacturers’ instructions.

### Assessment of Bcl2, bax, and caspase-3 protein expression levels

The expression levels of Bcl-2 and caspase-3 proteins in liver homogenates were assessed using ELISA kits from Cusabio Technology LLC (TX, USA) according to the manufacturer’s protocol. Additionally, a dedicated ELISA kit (MyBioSource Inc., CA, USA) was used to measure the expression level of Bax in liver homogenate according to the manufacturer’s protocol.

### Quantitative real-time polymerase chain reaction (qRT-PCR)

Total RNA from hepatic samples was extracted using the RNeasy mini kit (Qiagen, Hilden, Germany). 1-μg sample was transcribed into complementary DNA (cDNA) using the revert aid first strand cDNA synthesis kit (Thermo Scientific, Rockford, IL, USA) according to the manufacturers’ instructions. Quantitative real-time PCR (qRT-PCR) was conducted using the Hera Syber green RT-qPCR Kit (Willowfort, Birmingham, England) on a real-time thermal cycler (Thermo Fisher Scientific, Vantaa, Finland). Relative expression of CYP2E mRNA was calculated by the 2^−ΔΔCt^ method using GAPDH mRNA expression as the internal standard. The primers used were as follows: CYP2E1 forward, 5′-GTTGCCTTGCTTGTCTGGAT-3′ and reverse, 5′-AGGAATTGGGAAAGGTCCTG-3′ and GAPDH forward, 5′-CACTCTTCCACCTTCGATGC-3′ and reverse, 5′-CCCTGTTGCTGTAGCCGTAT-3′.

### Statistical analysis

All statistical analyses and graphing were conducted using Graphpad Prism V 5 (GraphPad Software Inc., San Diego, CA, USA). Mean ± standard error of mean (SEM) was used to express data. D'Agostino & Pearson omnibus normality test was used to test the normality of data. One-way analysis of variance (ANOVA) followed by Tukey–Kramer’s multiple comparisons post-hoc test were used to measure significant differences between groups. Non-parametric scoring data were compared using the Kruskal–Wallis test followed by Dunn’s tests for pair-wise comparisons. A p < 0.05 was considered significant for all tests.

## Results

### Effect of DCN and NAC on serum levels of ALT, AST, and LDH

**Fig. 1:**
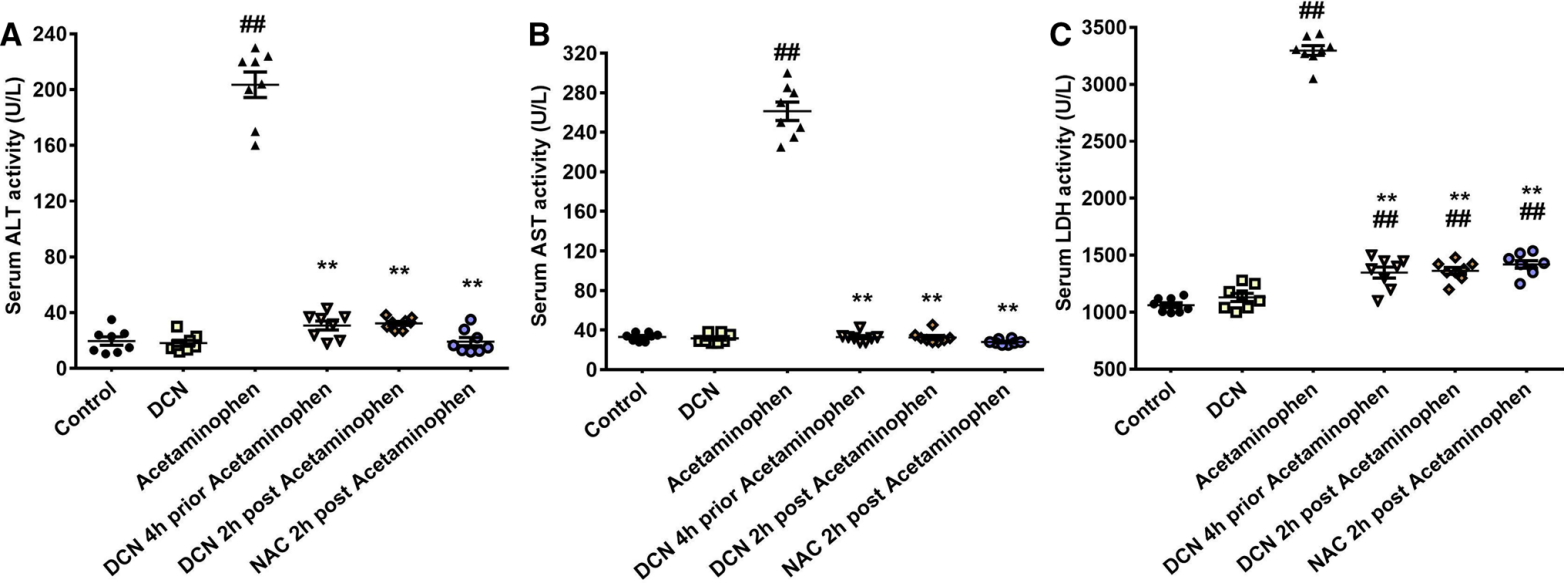
Effect of diacerein (DCN) and N-acetyl cysteine (NAC) on **A** alanine aminotransferase (ALT), **B** aspartate aminotransferase (AST), and **C** lactate dehydrogenase (LDH) serum levels in acetaminophen-injected mice. Data are expressed as mean ± SEM (n = 8). #p < 0.05, ##p < 0.01 compared to control group, *p < 0.05, **p < 0.01 compared to acetaminophen-injected group, ɸp < 0.05, ɸɸp < 0.01 compared to DCN 4 h prior to acetaminophen group, •p < 0.05, ••p < 0.01 compared to DCN 2 h post-acetaminophen group using one-way ANOVA followed by Tukey–Kramer multiple comparisons post hoc test

Administration of acetaminophen significantly (p < 0.01) elevated serum ALT activity by 10.36-fold, AST activity by 7.89-fold, and LDH activity by 3.10-fold compared to compared to the control group (Fig. 1A–C, respectively).

Administration of DCN 4 h prior to acetaminophen significantly (p < 0.01) reduced peak ALT activity by 84.86%, AST activity by 87.43%, and LDH activity by 59.11% compared to acetaminophen alone. Similarly, administration of DCN 2 h post-acetaminophen significantly (p < 0.01) reduced ALT activity by 84.10%, AST activity by 87.55%, and LDH activity by 58.65% compared to acetaminophen alone. Alternatively, DCN alone had no significant effect on serum ALT, AST, or LDH activity. Moreover, the suppressive effects of DCN on acetaminophen-induced ALT, AST, and LDH elevations were comparable to those of the standard antidote for acetaminophen overdose NAC (90.54, 89.33, and 56.93% reductions, respectively, compared to acetaminophen alone, p < 0.01).

### Effect of DCN and NAC on hepatic necroinflammation and infiltration of immune cells

**Fig. 2:**
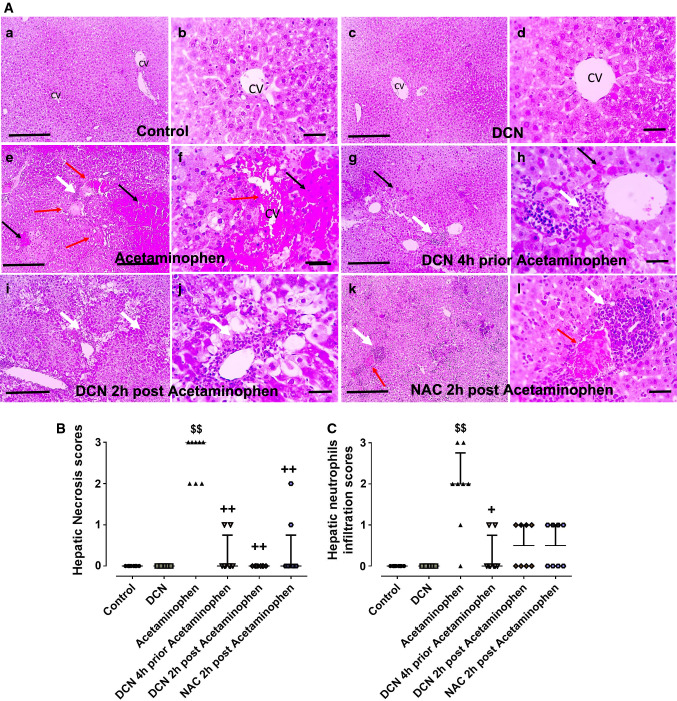
**A** Representative images of hematoxylin–eosin-stained liver sections showing the effect of diacerein (DCN) and N-acetyl cysteine (NAC) on acetaminophen-induced hepatic histopathological changes in mice (figures a, c, e, g, i and k: X 100, bar = 100 µm, figures b, d, f, h, j and l: X 400, bar = 50 µm). (a, b) Liver of control group showing normal arrangement of hepatic cords around central vein (CV) with normal sinusoids; (c, d) Liver of DCN group showing normal arrangement of hepatic cords around CV with normal sinusoids; (e, f) Liver of acetaminophen group showing confluent areas of necrosis (black arrows) with neutrophils infiltration (white arrows) around congested blood vessels (red arrows) and in area of hepatocytes necrosis; (g, h) Liver of DCN 4 h prior to acetaminophen group showing few cell deaths (black arrows) with few neutrophils infiltration (white arrows); (i, j) Liver of DCN 2 h post-acetaminophen group showed few neutrophil infiltration (white arrows) around CV; (k, l) Liver of NAC 2 h post acetaminophen-group showing smaller areas of necrosis with neutrophil infiltration (white arrows) around congested blood vessels (red arrows) and area of hepatocytes necrosis. **B** Scatter dot plots of the histopathological assessment of hepatic necrosis scores. **C** Scatter dot plots of the histopathological assessment of hepatic neutrophil infiltration scores. $p < 0.05, $$p < 0.01 compared to control group, +p < 0.05, ++p < 0.01 compared to acetaminophen group using Kruskal–Wallis followed by Dunn's Multiple comparison test

Hematoxylin and eosin-stained hepatic sections from the acetaminophen group revealed marked confluent areas of hepatocellular necrosis and neutrophils infiltration around both these necrotic areas and congested blood vessels (Fig. 2e, f) compared to hepatic sections from the control group (Fig. 2a, b) and the DCN group (Fig. 2c, d) that showed a normal arrangement of hepatic cords around central vein with normal sinusoids. Hepatic sections from mice administered DCN either 4 h prior to acetaminophen (Fig. 2g, h) or 2 h post-acetaminophen (Fig. 2i, j) contained only fewer dead cells and fewer infiltrating neutrophils than sections from mice administered acetaminophen alone. Similarly, hepatic sections from mice administered NAC 2 h post-acetaminophen contained only small areas of necrosis with neutrophil infiltration and a few congested blood vessels surrounded by infiltrating neutrophils (Fig. 2k, l).

The comparable efficacy of DCN compared to NAC was further confirmed by Semi-quantitative scoring of hepatic necrosis (Fig. 2B) and neutrophil infiltration (Fig. 2C). Both scores were significantly elevated in the acetaminophen alone group compared to the control group, and significantly reduced by DCN administration compared to the acetaminophen group whether administered 4 h before or 2 h after acetaminophen injection, and there were no significant differences compared to the NAC group.

**Fig. 3:**
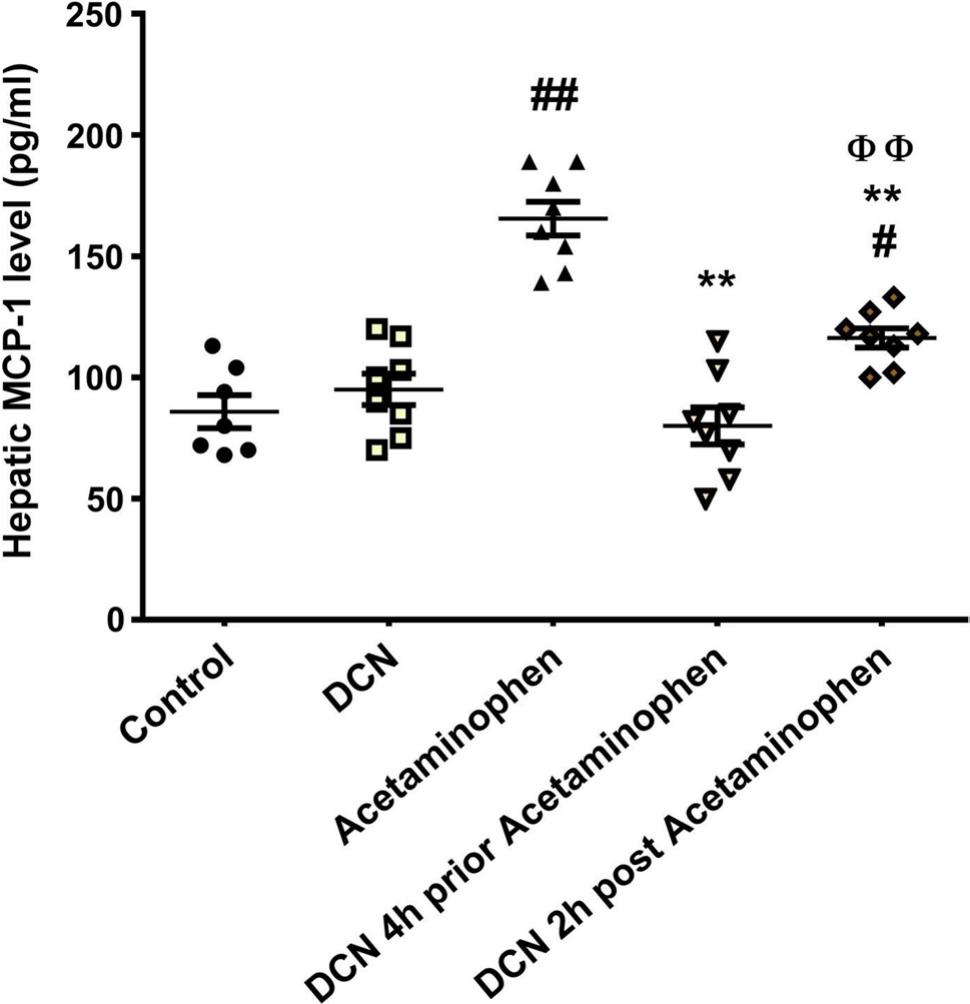
Effect of diacerein (DCN) on hepatic levels of monocyte chemoattractant protein-1 (MCP-1) in acetaminophen-injected mice. Data are expressed as mean ± SEM (n = 8). #p < 0.05, ##p < 0.01 compared to control group, *p < 0.05, **p < 0.01 compared to acetaminophen-injected group, ɸp < 0.05, ɸɸp < 0.01 compared to DCN 4 h prior to acetaminophen group, •p < 0.05, ••p < 0.01 compared to DCN 2 h post-acetaminophen group using one-way ANOVA followed by Tukey–Kramer multiple comparisons post hoc test

Acetaminophen injection also produced a significant (p < 0.01) 1.85-fold elevation in hepatic MCP-1 compared to the control group (Fig. 3), while DCN administration 4 h prior to acetaminophen significantly (p < 0.01) reduced this elevation by 51.66%. Administration of DCN 2 h after acetaminophen also produced a significant (p < 0.01) but smaller MCP-1 reduction of 29.72% compared to the acetaminophen alone group. Further, DCN alone had no effect on hepatic MCP-1 expression compared to the control group.

**Fig. 4:**
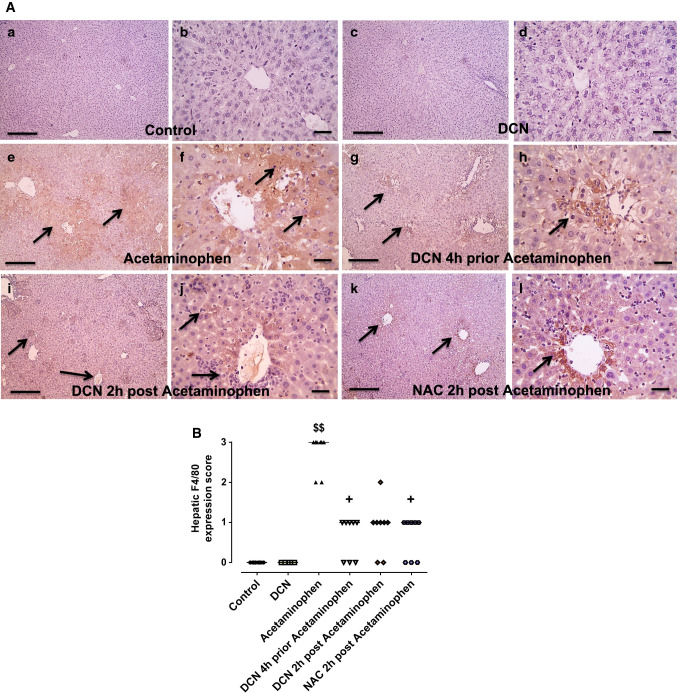
**A** Effect of diacerein (DCN) on hepatic F4/80 assessed by immunohistochemistry (figures a, c, e, g, i and k: X 100, bar = 100 µm, figures b, d, f, h, j and l: X 400, bar = 50 µm). (a, b) hepatic sections of control group showed negative staining; (c, d) hepatic sections of DCN group showed negative staining; (e, f) hepatic sections of acetaminophen group showed marked positive brown expression appears in hepatocytes (black arrows); (g, h) hepatic sections of DCN 4 h prior to acetaminophen group showed a significant reduction of F4/80 expression (black arrows); (i, j) hepatic sections of DCN 2 h post-acetaminophen group showed a significant reduction of F4/80 expression (black arrows); (k, l) hepatic sections of NAC 2 h post-acetaminophen-group showed a significant reduction of F4/80 expression (black arrows). **B** scatter dot plots of the immunohistopathological assessment of hepatic F4/80 scores. $$p < 0.01 compared to control group, +p < 0.05 compared to acetaminophen group using Kruskal–Wallis followed by Dunn's Multiple comparison test

We further investigated whether monocytes contribute to this acetaminophen-induced sterile inflammation by immunohistochemical analysis of the macrophage marker F4/80. Hepatic sections from both the control group [Fig. 4A(a), A(b)] and the DCN alone group [Fig. 4A(c), A(d)] exhibited few F4/80-positive cells, while such cells were numerous (brown stained) in the acetaminophen alone group [Fig. 4A(e), A(f)]. Consistent with H&E staining results, F4/80 expression was reduced by DCN whether administered 4 h prior to acetaminophen [Fig. 4A(g), A(h)] or 2 h after acetaminophen [Fig. 4A(i), A(j)], and expression did not appear to differ from the NAC group [Fig. 4A(k), A(l)].

Indeed, semi-quantitative scoring of F4/80 expression (Fig. 4B) showed a significant (p < 0.01) elevation in the acetaminophen group compared to the control group and significant (p < 0.05) reductions induced by DCN 4 h before, DCN 2 h after, and NAC 2 h after acetaminophen injection compared to the acetaminophen alone group.

### Effect of DCN on hepatic levels of the pro- and anti-apoptotic markers (Bcl2, bax, and caspase-3)

**Fig. 5:**
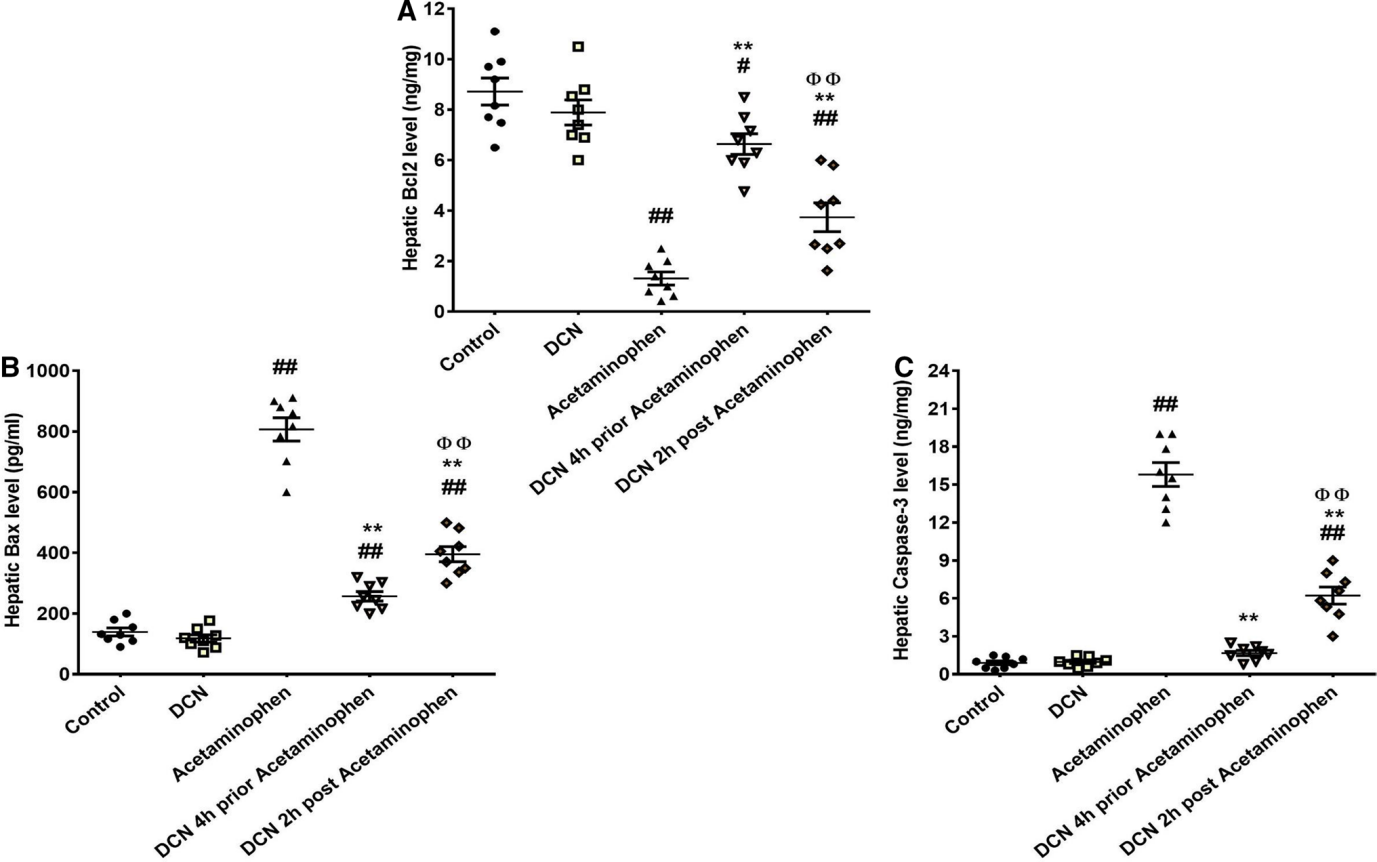
Effect of diacerein (DCN) on hepatic levels of **A** B-cell leukemia/lymphoma 2 (Bcl-2), **B** Bcl2 associated X (Bax), and **C** caspase-3 in acetaminophen-injected mice. Data are expressed as mean ± SEM (n = 8). #p < 0.05, ##p < 0.01 compared to control group, *p < 0.05, **p < 0.01 compared to acetaminophen group, ɸp < 0.05, ɸɸp < 0.01 compared to DCN 4 h prior to acetaminophen group, •p < 0.05, ••p < 0.01 compared to DCN 2 h post-acetaminophen group using one-way ANOVA followed by Tukey–Kramer multiple comparisons post hoc test

Hepatic expression of the anti-apoptotic protein Bcl2 was significantly (p < 0.01) reduced by 84.89% in the acetaminophen group compared to the control group (Fig. 5A), but significantly (p < 0.01) elevated by DCN, whether administered 4 h before acetaminophen (5.04-fold increase) or 2 h after acetaminophen (2.84-fold increase) compared to the acetaminophen group.

Conversely, hepatic expression of the pro-apoptotic proteins Bax (Fig. 5B) and caspase-3 (Fig. 5C) were significantly (p < 0.01) elevated by 5.80- and 17.56-fold, respectively, in the acetaminophen group compared to the control group, but significantly (p < 0.01) reduced by DCN administration 4 h before acetaminophen (68.17 and 89.38% decreases, respectively) and by DCN administration 2 h after acetaminophen (50.97 and 60.58% decreases, respectively) compared to acetaminophen alone. Thus, the pro-apoptotic condition induced by acetaminophen was effectively shifted back to an anti-apoptotic condition by DCN, especially by pretreatment.

### Effect of DCN and NAC on hepatic oxidative stress biomarkers and antioxidant enzymes

**Fig. 6:**
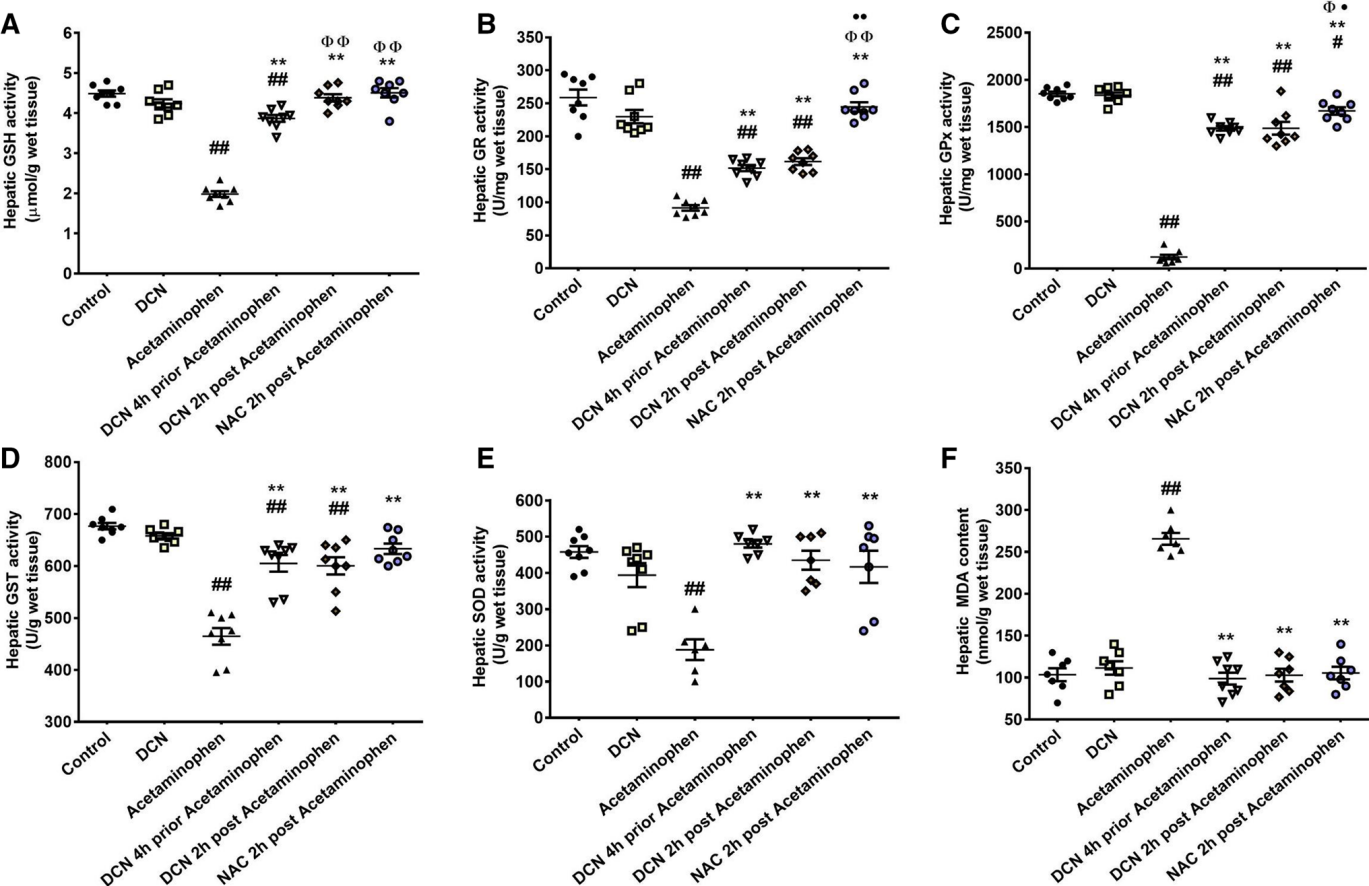
Effect of diacerein (DCN) and N-acetyl cysteine (NAC) on **A** reduced glutathione (GSH), **B** glutathione reductase (GR), **C** glutathione peroxidase (GPx), **D** glutathione-S-transferase (GST), **E** superoxide dismutase (SOD), and **F** malondialdehyde (MDA) in hepatic tissue of acetaminophen-injected mice. Data are expressed as mean ± SEM (n = 8). #p < 0.05, ##p < 0.01 compared to control group, *p < 0.05, **p < 0.01 compared to acetaminophen group, ɸp < 0.05, ɸɸp < 0.01 compared to DCN 4 h prior to acetaminophen group, •p < 0.05, ••p < 0.01 compared to DCN 2 h post-acetaminophen group using one-way ANOVA followed by Tukey–Kramer multiple comparisons post hoc test

Acetaminophen injection resulted in significant (p < 0.01) reductions in hepatic GSH concentration (by 55.79%) and in the activities of GR (by 64.47%), GPx (by 93.35%), GST (31.34%), and SOD (58.95%) compared to controls (Fig. 6A–E, respectively). This acetaminophen-induced reduction in cellular antioxidant capacity was associated with a significant (p < 0.01) 2.56-fold increase in MDA (Fig. 6F) compared to the control group.

While DCN alone had no significant effects on these antioxidant and oxidative stress biomarkers, administration of DCN 4 h before acetaminophen significantly (p < 0.01) elevated GSH concentration by 1.95-fold and the activities of GR by 1.65-fold, GPx by 12.08-fold, and GST by 1.30-fold compared to acetaminophen alone. Further, DCN pretreatment normalized SOD activity (i.e., to control levels) and significantly (p < 0.01) reduced MDA content by 62.78% compared to acetaminophen alone.

Similarly, administration of DCN 2 h after acetaminophen injection resulted in a significant (p < 0.01) elevation in the hepatic activities of GR (1.76-fold), GPx (12.09-fold), GST (1.29-fold), and SOD (2.31-fold) compared to acetaminophen alone. Administration of the standard antidote NAC 2 h after acetaminophen injection also restored hepatic levels of these antioxidants and oxidative stress markers to near control group levels.

### Effect of DCN on gene expression of CYP2E1in the liver

**Fig. 7:**
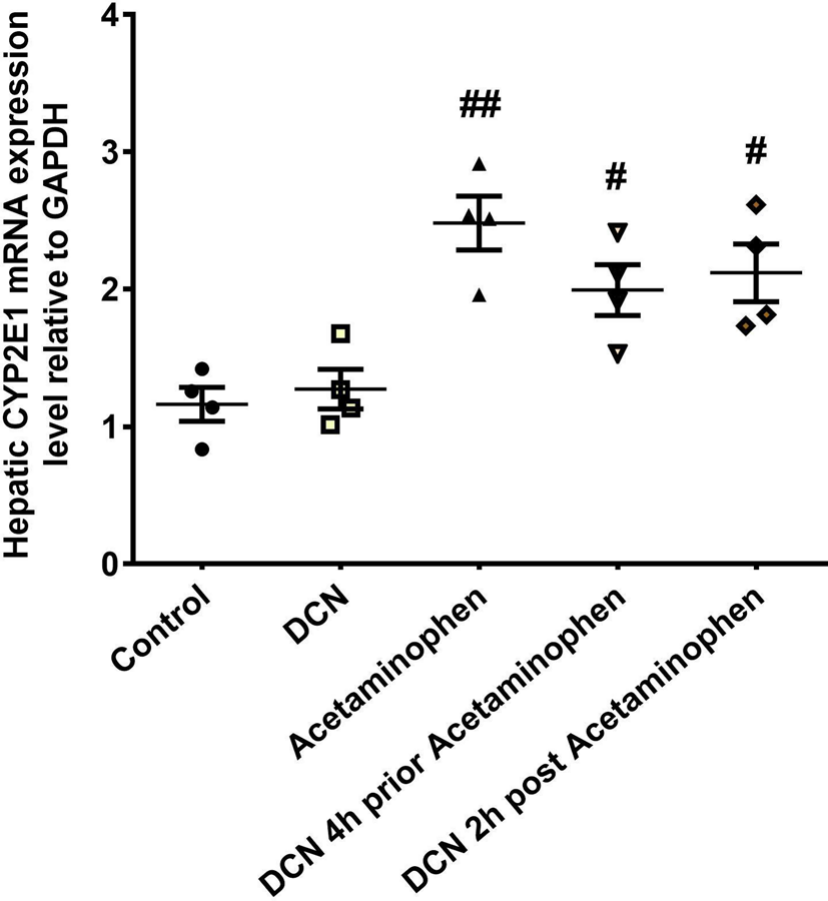
Effect of DCN on mRNA expression of CYP2E1 in hepatic tissue of acetaminophen- injected mice. Data are expressed as mean ± SEM (n = 4). CYP2E1: cytochrome P450 2E1, GAPDH: glyceraldehyde-3-phosphate dehydrogenase, #p < 0.05, ##p < 0.01 compared to control group using one-way ANOVA followed by Tukey–Kramer multiple comparisons post hoc test

The mRNA expression level of CYP2E1 mRNA in liver was significantly (p < 0.05) increased by 2.13-fold in the acetaminophen group compared to the control group. Treatment with DCN 4 h before acetaminophen reduced hepatic expression of CYP2E1 mRNA by 19.62% and treatment 2 h after acetaminophen by 14.59%, but these decreases did not reach statistical significance (p > 0.05) compared to the acetaminophen group (Fig. 7).

### Effect of DCN on hepatic TNF‐α level and NF-κB p65 expression

**Fig. 8:**
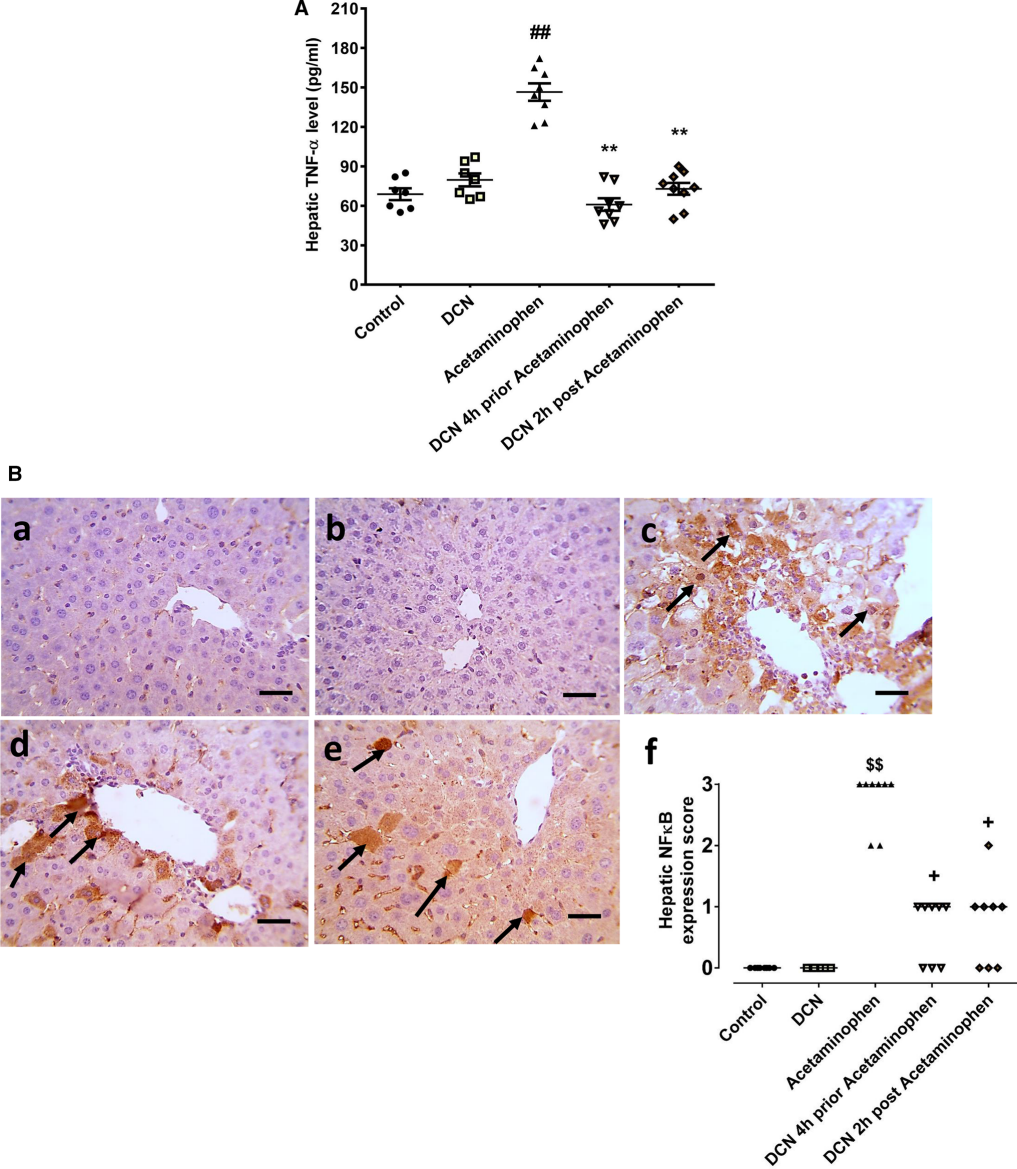
**A** Effect of diacerein (DCN) on hepatic tumor necrosis factor alpha (TNF-α) level. Data are expressed as mean ± SEM (n = 8). #p < 0.05, ##p < 0.01 compared to control group, *p < 0.05, **p < 0.01 compared to acetaminophen group, ɸp < 0.05, ɸɸp < 0.01 compared to DCN 4 h prior to acetaminophen group, •p < 0.05, ••p < 0.01 compared to DCN 2 h post-acetaminophen group using one-way ANOVA followed by Tukey–Kramer multiple comparisons post hoc test. **B** Effect of diacerein (DCN) on hepatic nuclear factor kappa-B p65 subunit (NF-κB p65) expression assessed by immunohistochemistry (X 400, bar = 50 µm); (a) hepatic sections of control group showing negative staining; (b) hepatic sections of DCN group showing negative staining; (c) hepatic sections of acetaminophen group showing marked positive brown nuclear and cytoplasmic expressions in hepatocytes (black arrows) associated with area of lesions around central veins; (d) hepatic sections of DCN 4 h prior to acetaminophen group showing a significant reduction of NF-κB p65 nuclear and cytoplasmic expressions (black arrows); (e) hepatic sections of DCN 2 h post-acetaminophen group showing a significant reduction of NF-κB p65 nuclear and cytoplasmic expressions (black arrows), and (f) scatter dot plots of the immunohistopathological assessment of hepatic NF-κB p65 score. $p < 0.05, $$p < 0.01 compared to control group, +p < 0.05, ++p < 0.01 compared to acetaminophen group using Kruskal–Wallis followed by Dunn's Multiple comparison test

Compared to the control group, acetaminophen administration significantly (p < 0.01) increased hepatic TNF-α by 2.07-fold (Fig. 8A), while DCN treatment 4 h before and 2 h after acetaminophen injection significantly (p < 0.01) reduced hepatic TNF-α by 58.36 and 50.24%, respectively, compared to acetaminophen alone. Injection of DCN alone had no effect on hepatic TNF-α.

Immunostaining revealed that NF-κB p65 was only sparsely expressed by liver sections from control and DCN group mice [Fig. 8B(a), B(b), respectively] but robustly expressed in both the nucleus and cytoplasm of hepatocytes (brown staining) within acetaminophen-induced lesions surrounding the central vein [Fig. 8B(c)]. Thus, acetaminophen-induced liver pathology was associated with the upregulation and nuclear translocation of this stress response transcription factor. Conversely, DCN administered either 4 h prior to acetaminophen injection [Fig. 8B(d)] or 2 h after acetaminophen injection [Fig. 8B(e)] reduced NF-κB p65 immunoexpression.

Semi-quantitative scoring of NF-κB p65 expression revealed a significant elevation in the acetaminophen group compared to the control group and significantly reductions in both DNC co-treatment groups compared to the acetaminophen group [Fig. 8B(f)].

### Effect of DCN on expression level of NLRP3, caspase-1, and IL-1β in the liver

**Fig. 9:**
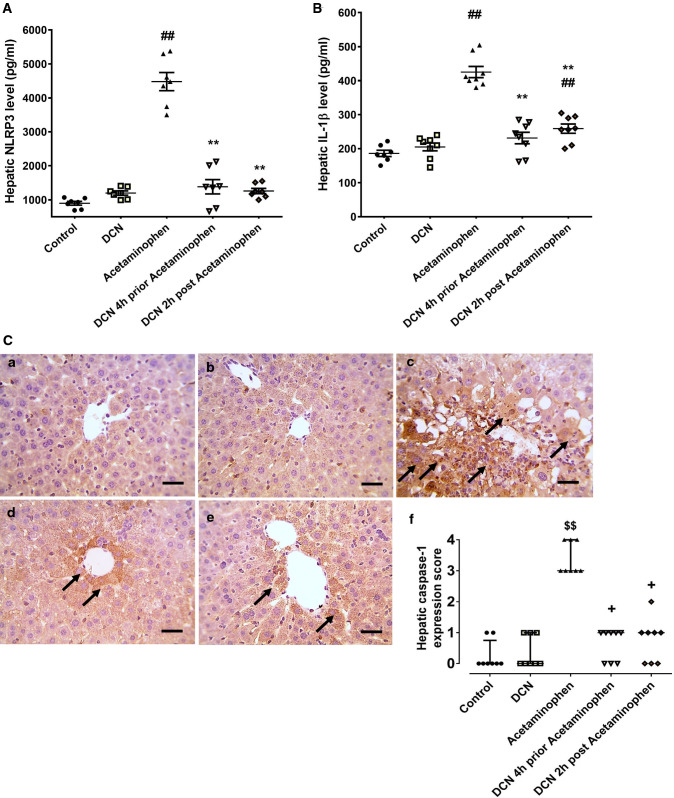
Effect of diacerein (DCN) on **A** NLR family pyrin domain containing 3 (NLRP3) and **B** Interlukin-1 beta (IL-1β) levels in hepatic tissue of acetaminophen-injected mice. Data are expressed as mean ± SEM (n = 8). #p < 0.05, ##p < 0.01 compared to control group, *p < 0.05, **p < 0.01 compared to acetaminophen-injected group, ɸp < 0.05, ɸɸp < 0.01 compared to DCN 4 h prior to acetaminophen group, •p < 0.05, ••p < 0.01 compared to DCN 2 h post-acetaminophen group using one-way ANOVA followed by Tukey–Kramer multiple comparisons post hoc test. **C** Effect of diacerein (DCN) on hepatic caspase-1 assessed by immunohistochemistry (X 400, bar = 50 µm); (a) hepatic sections of control group showing negative staining; (b) hepatic sections of DCN group showing negative staining; (c) hepatic sections of acetaminophen group showing marked positive brown cytoplasmic expression in hepatocytes (black arrows) associated with area of lesions around central veins; (d) hepatic sections of DCN 4 h prior to acetaminophen group showing a significant reduction of caspase-1 cytoplasmic expression (black arrows); (e) hepatic sections of DCN 2 h post-acetaminophen group showing a significant reduction of caspase-1 cytoplasmic expression (black arrows), and (f) scatter dot plots of the immunohistopathological assessment of hepatic caspase-1 scores. $p < 0.05, $$p < 0.01 compared to control group, +p < 0.05, ++p < 0.01 compared to compared to acetaminophen group using Kruskal–Wallis followed by Dunn's Multiple comparison test

Acetaminophen injection produced a significant (p < 0.01) 5.00-fold elevation in hepatic NLRP3 (Fig. 9A) and a 2.29-fold elevation in IL-1β (Fig. 9B) compared to the control group, while injection of DCN 4 h before acetaminophen significantly (p < 0.01) reduced these levels by 69.17 and 45.64%, respectively, and DCN injection 2 h after acetaminophen reduced these levels by 71.87 and 39.13%, respectively, compared to the acetaminophen group. Again, DCN injection alone had no effect on NLRP3 or IL-1β expression in the liver.

Liver sections from control [Fig. 9C(a)] and DCN alone groups [Fig. 9C(b)] showed little immunostaining for caspase-1, while immunoexpression was abundant in hepatocytes associated with acetaminophen-induced lesions around the central hepatic vein [Fig. 9C(c)]. Immunoexpression of caspase-1 in hepatocyte cytoplasm was substantially reduced by DCN injection 4 h after acetaminophen injection [Fig. 9C(d)] and by DCN injection 2 h after acetaminophen injection [Fig. 9C(e)].

Semi-quantitative scoring of caspase-1 expression [Fig. 9C(f)] revealed a significant elevation following acetaminophen injection compared to the control group, while administration of DCN 4 h before or 2 h after acetaminophen injection significantly counteracted this elevation compared to the acetaminophen group.

### Effect of DCN on hepatic IL-4 and IL-10 levels

**Fig. 10:**
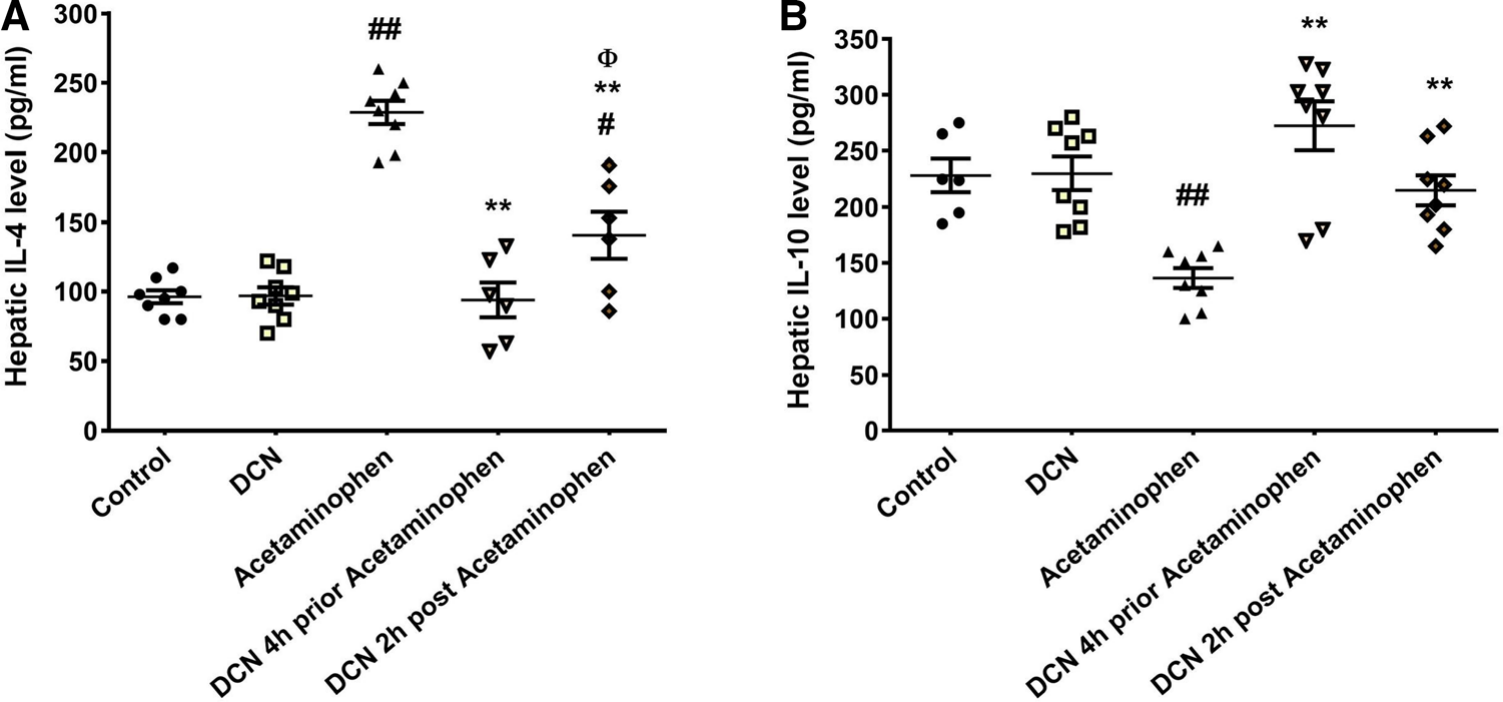
Effect of diacerein (DCN) on hepatic levels of **A** interleukin (IL)-4, and **B** IL-10 in acetaminophen-injected mice. Data are expressed as mean ± SEM (n = 8). #p < 0.05, ##p < 0.01 compared to control group, *p < 0.05, **p < 0.01 compared to acetaminophen group, ɸp < 0.05, ɸɸp < 0.01 compared to DCN 4 h prior to acetaminophen group, •p < 0.05, ••p < 0.01 compared to DCN 2 h post-acetaminophen group using one-way ANOVA followed by Tukey–Kramer multiple comparisons post hoc test

Finally, acetaminophen injection also induced a significant (p < 0.01) 2.31-fold elevation in hepatic IL-4 (Fig. 10A) compared to the control group, while administration of DCN 4 h before acetaminophen significantly (p < 0.01) decreased hepatic IL-4 by 58.91%. Administration of DCN 2 h after acetaminophen injection also produced a significant (p < 0.01) but smaller IL-4 reduction of 38.50% compared to the acetaminophen group.

Conversely, acetaminophen administration significantly (p < 0.01) reduced hepatic IL-10 by 40.21% compared to the control group (Fig. 10B), while DCN treatment 4 h before acetaminophen injection significantly (p < 0.01) increased IL-10 level by 1.99-fold and treatment 2 h after acetaminophen injection increased IL-10 by 1.57-fold compared to the acetaminophen group. Injection of DCN had no significant effects on IL-4 and IL-10 levels compared to the control group.

## Discussion

Oxidative stress and associated mitochondrial dysfunction due to GSH depletion is the central pathogenic pathway underlying acetaminophen hepatotoxicity (Jaeschke and McGill 2015). The cysteine prodrug NAC is still the first line therapy used to counteract NAPQI-induced GSH depletion and restore hepatic redox balance following acetaminophen overdose.

Unfortunately, NAC has a relatively narrow therapeutic window and in many patients, especially the elderly, liver transplantation is the only option to improve survival. Therefore, new therapeutic options for acetaminophen hepatotoxicity that ensure safety and efficacy are required (Bateman and Dear 2019; Chang et al. 2020).

Previous studies have reported that the efficacy of natural substances for preventing acetaminophen hepatotoxicity, but these agents have demonstrated limited efficacy as curative therapies (Jin et al. 2017; Chang et al. 2020). Accordingly, the current study assessed the preventive and curative effects of DCN and elucidated its potential underlying mechanisms. Indeed, DCN effectively reduced acetaminophen hepatotoxicity whether administered before or after acetaminophen injection via anti-oxidant, anti-inflammatory, anti-necrotic, and anti-apoptotic mechanisms. Further, DCN alone had no deleterious side-effects at the chosen dose.

Diacerein is included by many rheumatology society guidelines as a therapeutic option for osteoarthritis, including those of the European League Against Rheumatism (EULAR) (Jordan et al. 2003). Initially, the Pharmacovigilance Risk Assessment Committee (PRAC) of the European Medicines Agency (EMA) recommended restricting DCN use due to rare reports of severe diarrhea and elevated serum liver enzymes. However, following re-examination in July 2014, the PRAC/EMA confirmed the safety profile of DCN and stated that its benefits outweigh its known risks for the treatment of osteoarthritis (Pavelka et al. 2016). Subsequent preclinical studies and clinical trials have confirmed the safety of DCN and demonstrated additional promising pharmacological effects, including hepatoprotection (Bu et al. 2018; Leite et al. 2019; Pelletier et al. 2020)**.**

Acetaminophen overdose initiates a vicious cycle of oxidative stress and mitochondrial dysfunction that ultimately results in hepatocellular necrosis. Hepatocyte necrosis is manifested by elevated serum concentrations of ALT, AST, and LDH (Uysal et al. 2016; Yoon et al. 2016). Diacerein both prevented and reversed these increases, consistent with histopathological findings showing marked suppression of acetaminophen-induced hepatocellular necrosis and inflammation.

While hepatocellular necrosis is the main mechanism of hepatocyte death under acetaminophen overdose, apoptotic death may also contribute (Jaeschke et al. 2018). Consistent with this notion, acetaminophen injection significantly decreased the hepatic level of anti-apoptotic Bcl-2 and increased hepatic levels of both the pro-apoptotic Bax and the apoptotic effector caspase-3. In addition to prevention of necrosis, DCN likely prevented apoptosis as evidence by reversal of these changes in Bcl-2, Bax, and caspase-3 expression.

N-acetylcysteine reverses the deleterious effects of acetaminophen by restoring endogenous GSH depleted by the acetaminophen metabolite NAPQI. Restoration of GSH in turn sustains the activities of GSH-dependent enzymes such of GR, GPx, and GST (Yan et al. 2008). In the absence of sufficient GSH antioxidant capacity, reactive oxygen species such as superoxide produced by the mitochondrial electron transport chain accumulate. This superoxide is converted by SOD to hydrogen peroxide, which in the presence of iron forms highly reactive hydroxyl radicals. Hydroxyl radicals cause oxidative damage to cellular macromolecules, including peroxidation of membrane lipids, which among other effects disrupts cellular and mitochondrial membrane permeability (Laine et al. 2009; McGill et al. 2012; Amin et al. 2017).

In the current study, acetaminophen significantly decreased hepatic GSH level as well as GR, GPx, GST, and SOD activities, and increased hepatic content of the lipid peroxidation biomarker MDA. Notably, DCN both prevented and reversed this acetaminophen-induced reduction in hepatic antioxidant capacity but did not reverse acetaminophen-induced upregulation of hepatic CYP2E1 mRNA, which encodes the enzyme responsible for toxic NAPQI generation. Accordingly, DCN appears to act mainly by sustaining the antioxidant response against NAPQI rather than by reducing NAPQI production. Further, these effects on GSH and GSH-dependent enzymes were comparable to those of NAC.

Another important factor in the pathogenesis of acetaminophen hepatotoxicity is sterile inflammation secondary to oxidative stress and necrotic cell death (Woolbright and Jaeschke 2017). Mitochondrial oxidative stress can trigger signaling cascades that ultimately lead to the production of pro-inflammatory cytokines such as TNF-α (Blazka et al. 1995). Targeting TNF-α represents a promising strategy to limit hepatotoxicity as it was reported that serum ALT level was increased more than 50% under acetaminophen overdose by exogenous TNF-α (Ishida et al. 2004; Gandhi et al. 2010). In the current study as well, acetaminophen overdose markedly elevated while DCN normalized hepatic TNF-α whether administered before or after acetaminophen.

Furthermore, acetaminophen overdose increased the hepatic expression and nuclear translocation of NF-κB, a stress-associated transcription factor that controls various genes involved in the oxidative stress response, inflammation, and cytoprotection (Posadas et al. 2012; Lingappan 2018). Following acetaminophen overdose, NF-κB induces upregulation of the pro-inflammatory cytokines such as TNF-α and IL-1β (Dambach et al. 2006; Liu et al. 2017). In this study, DCN prevented and reversed NF-κB overexpression and nuclear translocation as well as pro-inflammatory cytokine elevation, indicating that DCN protects liver function by mitigating intracellular oxidative as well as stress inflammatory cytokine signaling.

Hepatocellular necrosis is followed by the release of DAMPs that may induce sterile inflammation via activation of the NLRP3 inflammasome (Krenkel et al. 2014). This inflammasome activation has been implicated in the recruitment of neutrophils and monocytes during the progression of acetaminophen hepatotoxicity (Woolbright and Jaeschke 2017). Activation of NLRP3 leads to the cleavage of pro-IL-1β to active IL-1β via activated caspase-1, and IL-1β was reported to promote the mass infiltration of monocytes and neutrophils into the liver, which further aggravates hepatocellular injury and necrosis during acetaminophen overdose (Martinon et al. 2002; Cover et al. 2006; Ju 2012; Seok et al., 2021). Furthermore, mice lacking the capacity to activate the NLRP3 inflammasome and produce active IL-1β exhibited less severe acetaminophen-induced liver injury and inflammation (Ishibe et al. 2009; Szabo and Csak 2012). Recently, DCN was reported to protect against cadmium-induced testicular toxicity in rats by downregulating of NLRP3/caspase-1/ IL-1β signaling pathway-mediated inflammation and apoptosis (Fouad et al. 2020).

In the present study as well, DCN markedly inhibited acetaminophen-induced activation of this NLRP3 signaling axis whether injected before or after acetaminophen. The NLRP3**/**caspase-1/IL-1β axis may be also linked to apoptosis, as elevated expression of Bcl-2 protein attenuated NLRP3 activation (Dempsey 2012; Fouad et al. 2020). Also, recent findings suggest that apoptosis drives NLRP3 inflammasome activation under inflammatory conditions (Tsuchiya 2020).

In addition, assessment of neutrophil infiltration and hepatic expression of F4/80 positive cells clearly showed that the increased number of infiltrating neutrophils and monocytes in liver following acetaminophen injection was counteracted by DCN. Moreover, these results were confirmed by an assay showing that DCN also reduced the acetaminophen-induced elevation in hepatic MCP-1 level. MCP-1 is a well-recognized and major immune cell attractant that contributes to tissue injury by amplifying the initial inflammatory response (Mandrekar et al. 2011).

Immune cells, in particular the T helper (Th)-2 cell subtype of CD4 + Th cells and natural killer T cells, act as critical regulators of experimentally induced hepatic injury by releasing both pro- and anti-inflammatory effector cytokines, including IL-4 and IL-10. However, it remains controversial whether IL-4 contributes to acetaminophen hepatotoxicity (Lee et al. 2012; Krenkel et al. 2014; Elshal et al., 2021). It was reported that IL-4 induces expression of downstream factors such as MCP-1 that recruit monocytes and neutrophils to injured areas and hence sustain the inflammatory vicious cycle (Ratthé et al. 2009; Chen et al. 2011). It was reported that infiltrating neutrophils can regulate subsequent monocyte infiltration (Ishida et al. 2006). Ratthé et al (2009) reported that IL-4 increased the recruitment of neutrophils by 60% and monocytes by 40% in a mouse model (Ratthé et al. 2009).

Here we show that acetaminophen hepatotoxicity is associated with infiltration of leukocytes, particularly neutrophils and monocytes, and confirm that acetaminophen overdose can increase hepatic IL-4 level, and that DCN can significantly decrease this elevation when injected before or after acetaminophen. Therefore, targeting IL-4 may be another therapeutic mechanism by which DCN prevents or reverses acetaminophen hepatotoxicity. Conversely, acetaminophen suppressed and DCN normalized expression of the anti-inflammatory cytokine IL-10, in accord with studies reporting that IL-10 negatively regulates the production of pro-inflammatory cytokines such as TNF-α and inhibits the expression of NF-κB during acute hepatitis, including acetaminophen hepatotoxicity (Gardner et al. 2002; Gaddi et al. 2012; Dong et al. 2019).

**Fig. 11:**
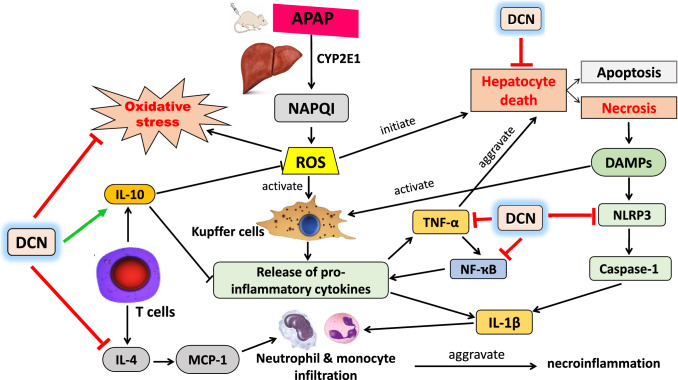
Schematic representation of the proposed underlying mechanisms of DCN against acetaminophen-induced hepatotoxicity in mice

In conclusion, DCN both prevents and reverses acetaminophen hepatotoxicity in mice by sustaining cellular oxidant capacity, thereby preventing oxidative stress, mitochondrial dysfunction, necrosis, sterile inflammation, and apoptosis. These effects were mediated by downregulation of the NLRP3/caspase-1/IL-1β, IL-4/MCP-1, and TNF-α/NF-κB pro-inflammatory signaling pathways. In addition, DCN mitigated second inflammatory responses by increasing hepatic IL-10 production (Fig. 11).
